# The Influence of Brief Outing and Temporary Fostering Programs on Shelter Dog Welfare

**DOI:** 10.3390/ani13223528

**Published:** 2023-11-15

**Authors:** Lisa M. Gunter, Emily M. Blade, Rachel J. Gilchrist, Betsy J. Nixon, Jenifer L. Reed, Joanna M. Platzer, Ingrid C. Wurpts, Erica N. Feuerbacher, Clive D. L. Wynne

**Affiliations:** 1 Department of Psychology, Arizona State University, Tempe, AZ 85281, USA; emily@shelteranimalscount.org (E.M.B.); rgilchri@asu.edu (R.J.G.); cwynne1@asu.edu (C.D.L.W.); 2 School of Animal Sciences, Virginia Polytechnic Institute and State University (Virginia Tech), Blacksburg, VA 24061, USA; jtay6366@gmail.com (J.L.R.); jplatzer@vt.edu (J.M.P.); enf007@vt.edu (E.N.F.); 3 State Farm Insurance, Phoenix, AZ 85016, USA; ingrid.wurpts@gmail.com

**Keywords:** dogs, animal shelter, human–animal interaction, welfare, adoption

## Abstract

**Simple Summary:**

Animal shelters can be stressful for dogs, but human interaction can improve their experience. While at the shelter, dogs’ stress can be reduced by spending time with a person outside of their kennel as can leaving the shelter for an overnight or longer stay in a foster home. In this study, we analyzed data of 1955 dogs from 51 animal shelters that went on an outing of a few hours or fostering stay of 1–2 nights, and 25,946 dogs that resided at these shelters but did not experience these interventions (controls). We found that outings and temporary fostering stays increased dogs’ likelihood of adoption by five and over 14 times, respectively. While dogs that experienced these interventions spent longer in the shelter awaiting adoption as compared to non-intervention dogs, this difference in length of stay was present prior to the dogs’ outings and fostering stays. We found that shelters’ intervention programs were more successful when members of the community were more involved in providing these experiences (in contrast to volunteers and staff) as well as when these organizations had more resources. Animal shelters should consider implementing brief outing and temporary fostering programs to improve the welfare of shelter-living dogs.

**Abstract:**

Human interaction is one of the most consistently effective interventions that can improve the welfare of shelter-living dogs. Time out of the kennel with a person has been shown to reduce physiological measures of stress as can leaving the shelter for a night or more in a foster home. In this study, we assessed the effects of brief outings and temporary fostering stays on dogs’ length of stay and outcomes. In total, we analyzed data of 1955 dogs from 51 animal shelters that received these interventions as well as 25,946 dogs residing at these shelters that served as our controls. We found that brief outings and temporary fostering stays increased dogs’ likelihood of adoption by 5.0 and 14.3 times, respectively. While their lengths of stay were longer in comparison to control dogs, this difference was present prior to the intervention. Additionally, we found that these programs were more successful when greater percentages of community members (as compared to volunteers and staff) were involved in caregiving as well as when programs were implemented by better-resourced shelters. As such, animal welfare organizations should consider implementing these fostering programs as evidence-based best practices that can positively impact the outcomes of shelter dogs.

## 1. Introduction

Millions of dogs enter animal sheltering facilities across the United States each year [1]. While a dog’s temporary stay in the shelter is likely stressful when compared to life in a home [2,3,4], the outcomes of dogs that experience this fate have improved considerably over the past two decades [5,6]. Overall, dogs are more often reclaimed by their owners, adopted, or transferred to other shelters for placement, while canine euthanasia is occurring less often [7,8,9].

This improvement in live outcomes provides an opportunity to further explore the welfare of dogs as they reside in animal shelters. Mellor and Reid [10] describe animal welfare as a state of being, both mental and physical, that an animal experiences. In total, these experiences are subjective within the animal, and an animal’s welfare or well-being is the integration of these experiences. Within the literature, scientists have focused their evaluations of welfare on the animal’s biological functioning, its affective state, and how closely its living situation matches its natural state of being [11].

Because a dog’s residency in the animal shelter is temporary, we can measure its welfare in two ways: proximally and distally. The proximal evaluation of a dog’s welfare is concerned with what the dog is currently experiencing. This approach is closest in perspective to how applied scientists measure the welfare of other captive animals in order to provide them with optimal care [12]. Here, measurements of health, physiology, behavior, and cognition inform welfare assessment.

Animal-based measurements, such as a dog’s body condition score, skin condition, and overall cleanliness can inform welfare assessment in the shelter [13,14]. Cortisol, a hormone involved in animals’ stress response system, is elevated when dogs are living in the shelter as compared to a home [3,4,15,16,17]. In the animal shelter, dogs rest and sleep less compared to when they are temporarily staying or living in a home [3,16,18]. Furthermore, when shelter dogs sleep more during the daytime in the shelter, they demonstrate a positive bias during a spatial cognitive bias task, an indication of better welfare [19].

Human interaction is one of the most well-studied interventions in animal sheltering. Specifically, dogs spending time with a person outside of the kennel has been consistently shown to improve dogs’ proximal welfare by reducing measures of stress and improving their behavior (for an in-depth review, see Gunter and Feuerbacher [20]). Gunter et al. [16] found that stays of one or two nights in a home reduce dogs’ cortisol levels and increase their longest bouts of rest. Ferhinger [15] found a similar effect on cortisol when dogs were provided with three days of fostering. Conversely, brief outings with a person into the community have been shown to increase dogs’ cortisol levels, even after accounting for their activity [19]. Regardless of their direction of impact, the effects of out-of-shelter human-interaction interventions are short-lived. Upon return to the shelter, dogs typically return to baseline cortisol and activity levels [16,21].

Measuring dogs’ distal welfare involves the ultimate goal of animal sheltering: dogs permanently leaving the shelter and living in a human home. From this perspective, dogs’ lengths of stay and outcomes are evaluated to assess welfare. Many characteristics of the dog can influence how long it stays in the shelter and its adoption likelihood, but these qualities are often immutable, such as the dogs’ morphology or how it arrived to the shelter [22]. Only a handful of empirically evaluated adoption interventions have been shown to reduce dogs’ time in the shelter or increase the possibility of a positive outcome. These include the facilitation of a dog’s adoption by a foster caregiver [23], altering the dog’s behavior with potential adopters [24], and removing labels used to describe a dog’s visually-identified breed [25,26].

Thus, while the proximal effects of brief outings and temporary fostering on shelter dog welfare have been explored, what is less understood is whether dogs’ length of stay in the care of the shelter or their likelihood of adoption are altered by these interventions. Generally, canine foster caregiving has been shown to improve dogs’ distal welfare [27,28]. Thus, in the present study, we hypothesized that both brief outings and temporary fostering would result in reduced lengths of stay and better outcomes for shelter dogs, as compared to dogs living in the shelter during the same time period that did not experience these interventions.

## 2. Materials and Methods

### 2.1. Shelter Recruitment and Enrollment

Animal welfare organizations operating in the United States were contacted for enrollment in this study via websites, social media announcements, and targeted email invitations. After confirming that an organization’s dogs lived in a facility for most of their sheltering stay (as opposed to a foster caregiver’s home), we conducted interviews with these organizations. Prospective organizations needed to collect data about their program in order to participate in the study.

Additionally, information about the shelters’ existing brief outing and temporary fostering programs was used as criteria for enrollment. For shelters participating in the brief outing component of the study, these organizations needed to be (1) without a brief outing program or (2) their program was experienced by an average of less than 10% of their canine population. For shelters enrolling in the study’s temporary fostering component, these organizations needed to already have a brief outing program in place that provided at least 10% or more of their canine population with this activity in order to participate. Additionally, they either had (1) no temporary fostering program in place or (2) an existing program that, on average, served less than 10% of their canine population. Shelters with existing brief outing and temporary fostering programs, in which at least 10% or more of their dog populations participated, were not eligible to enroll in the study.

During the interview process, demographic information about the shelters was collected, including their admission type (i.e., open, managed, or limited) and organization type (i.e., municipal, private nonprofit, or private nonprofit with municipal contracts). Open admission was defined as shelters with unrestricted animal intake from the areas they served, while those with managed admission controlled the arrival of incoming animals. Limited-admission shelters restricted the animals accepted into their care [29]. Information was also gathered about the types of fostering opportunities offered by each organization and their adoption policies.

Shelters also provided animal intake and budgetary data for the year prior to the study. Using these data, we calculated each shelter’s live release rate (dividing the sum of all live outcomes for dogs by total dog outcomes [30]) as well as the shelter’s resource level (as previously described in Gunter et al. [28]).

### 2.2. Programmatic Training and Support

After study enrollment, staff at participating animal shelters attended a training program, provided by Maddie’s Fund (Pleasanton, CA, USA), which discussed procedures for implementing and operating brief outing and temporary fostering programs. Once shelter staff completed this training, members of the research team met every other week with staff in video calls and engaged in email correspondence, assisting the shelter in the development of either a brief outing or temporary fostering program.

This initial support culminated in the launch of the shelter’s program, which occurred no later than 45 days after attending the training program. During the program’s launch, a member of the research team provided on-site assistance to the shelter for 3 days, and afterward, the team continued remote bi-weekly contact and email correspondence with each organization until data collection was complete. Following the shelter’s final brief outing or temporary fostering stay, data collection continued for an additional 7 days to record the dogs’ outcomes.

### 2.3. Dogs

Shelter staff determined which dogs would participate in their shelter’s intervention; however, outings and stays needed to be with dogs six months of age or older and residing at the shelter at the time of participation. Shelters provided data about each dog that experienced the intervention, including their age, weight, sex, intake date and type, outcome date and type, and adoption status (i.e., available for adoption or not available due to medical, behavioral, or other reasons) on the date of their intervention experience. Using dogs’ intake and outcome data, as well as the duration of their outing or stay, we were able to calculate their lengths of stay prior to and after the intervention.

### 2.4. Brief Outings and Temporary Fostering Stays

Brief outings were conducted off the property of the animal shelter with at least one person (e.g., staff, volunteer, or community member). Similar to the brief outings described by Gunter et al. [21], outings lasted approximately 1–4 h. Off-site excursions to shelter-facilitated adoption events were not included. Temporary fostering stays were defined as dogs spending 1–2 nights in the home of a shelter staff person, volunteer, or member of the community, which has previously been described by Gunter et al. [16]. Although shelter dogs were sometimes placed into foster homes that included one or more resident dogs, shelter dogs were not placed together, except in instances where dogs were considered a bonded pair by shelter staff.

The duration of the intervention experiences and whether a dog bite occurred, either to a person or another dog, were also recorded.

### 2.5. Foster Caregivers

Shelter staff also collected data about the foster caregiver and their brief outing or temporary fostering experience, including the caregiver’s age, their relationship to the organization, the date and time that their outing or stay began and ended, and if they adopted their dog. From these data, we were able to calculate the total number of caregivers that participated at each shelter as well as the total number of foster experiences provided.

Additional information about the caregivers providing temporary foster care was gathered, including whether the person was previously involved with the shelter’s brief outing or temporary foster programs and the number of dogs residing in their home. If the caregiver had resident dogs, the shelter recorded the method of introduction between the resident and shelter dog (e.g., conducted at the shelter, at the caregiver’s home, or the dogs were separated during the fostering stay).

### 2.6. Intervention Impact and Program Performance

In order to evaluate the impacts of these interventions on shelter dog outcomes and length of stay, inventory reports were gathered about the dogs that resided in their shelters but did not participate in the intervention during the data collection period. These non-intervention dogs served as our study’s controls. The reports included dogs’ intake date and type, age, weight, sex, and outcome date and type and were obtained from shelter data management programs and other sources (e.g., cloud-based spreadsheets and paper records). Dogs in both the intervention and control conditions were either residing in the shelter prior to the launch of their intervention program or arrived during the data collection period. Due to data tracking and reporting limitations, comparison data on non-participating dogs were not available from Mendocino County Animal Control.

In an effort to evaluate the performance of these interventions amongst our participating shelters while accounting for differences in their canine intake, we ranked shelters on (1) the number of foster experiences provided during the data collection period, (2) the number of foster caregivers providing those experiences, and (3) the number of days needed for the shelter to enroll 40 dogs in the study, regardless of intervention type. This 40-dog-per-shelter sample size benchmark was used in order to reach an adequate number of participants based on previous research [16,21].

As such, programs that provided the most intervention experiences to dogs and had the most caregivers participating in their programs were ranked highest on those variables, while a shelter that needed the fewest days to collect their data was ranked higher than a shelter that took longer to reach the study’s sample size. Using each shelter’s rankings on these three variables, a summed ranking value was calculated. This overall rank was used to assess program performance in relation to characteristics of the shelter and its foster caregivers.

### 2.7. Data Analytic Approach

In the estimation of descriptive statistics during data analysis, we identified that many continuous variables (related to the shelters, dogs, interventions, caregivers, and program performance) were non-normally distributed. As such, means, measures of data variability (i.e., standard deviation, standard error, and confidence intervals), medians, and ranges are reported.

To estimate the difference in adoption rates between dogs who experienced brief outings versus temporary fostering stays, we used a chi-square goodness of fit test.

To understand the impacts of these interventions on adoption versus other outcomes (i.e., remained in care, transfer out, or euthanasia), we used two multinomial logistic regression models comparing dogs that received an intervention (outing or fostering stay) with control dogs (those that did not receive an intervention). Adoption served as the reference category for the dependent variable. We attempted to include shelter as a random effect in these models, but they failed to converge, so only models with fixed effects were employed.

In these regression analyses, relative risk ratios (RR) are reported for brief outings (BO/RR) and temporary fostering (TF/RR), which indicates the probability of an outcome for the intervention dogs compared to the probability of the same outcome for dogs that did not receive a brief outing or temporary fostering stay. As such, an RR value greater than one indicates how many times more likely that particular outcome is to occur for intervention dogs than dogs in the comparison group. With ratios of probabilities less than one, those RR values can be used as the divisor with one as the dividend to yield the outcome that is *X* times more likely to occur. Confidence intervals for relative risk ratios are reported alongside these values.

We used two linear regression models to estimate the effect of the intervention (brief outing or temporary fostering stay) on length of stay. Our dependent variable, length of stay in days, was log-transformed to account for its positive skew. In these analyses, dogs that were returned to their owners were excluded.

In both types of regression models, we entered dog-level covariates including their sex (i.e., male or female), age (in months), weight (in kilograms), number of times the intervention was experienced, and intake type (i.e., stray, cruelty/neglect, transfer in, owner surrender/return). Among dogs that were temporarily fostered, we estimated the additional effect of an intervention-level covariate: number of resident dogs in the caregiver’s home.

To explore factors related to the performance of intervention programs among our study shelters, we used an ordinary least squares regression model with intervention type (brief outing or temporary fostering) and shelter-related characteristics (i.e., organization and admission types, resource level, and proportions of volunteer and community caregivers). Our dependent variable, program performance, was a summed ranking value based on foster experiences, caregivers, and days of data collection.

All models were evaluated for data sparsity by cross-tabulating categorical independent variables with the categorical dependent variable. In instances where cell counts were at or near zero, groups (e.g., dogs remaining in the organization’s care at the shelter or in foster care) were combined when appropriate. For models utilizing continuous covariates (i.e., dog age and weight), we screened for outliers and capped or floored these variables. When dogs had more than one outing or fostering stay during data collection, associated logistic and OLS regression models were estimated using the dog’s earliest intervention experience in order to avoid an individual dog contributing multiple cases to our model estimations. All analyses were conducted in R.

## 3. Results

### 3.1. Descriptive Statistics

#### 3.1.1. Shelters

Between February 2019 and March 2020, we collected data with 51 US animal shelters about their brief outing and temporary fostering programs with each shelter. In total, 60 foster programs are represented in this sample (nine animal shelters participated in both interventions investigated in this study).

Live release rates (LRRs) for dogs varied across our shelters but were relatively high with an average rate of 91.9% (*SD* = 9.4%), ranging from 63 to 100% with a median of 95.6%. Shelters’ annual operating budgets for the year prior to the study varied considerably from USD 200,000 to over USD 23 million (*M* = USD 3,983,677, *Mdn* = USD 2,155,613, *SD* = USD 4,394,998).

The number of animals that shelters brought into their facilities each year differed by several fold. While the average number was 6569 animals (*Mdn* = 4879, *SD* = 7051), the intake of shelters ranged from 241 to 32,788 animals. On average, the proportion of dogs in these shelters accounted for roughly half of all animals (*M* = 50.4%, *SD* = 16.5) with a range of 27 to 100%, and a median of 47.8%.

Table 1 describes the average, median, and range of operating budgets and annual intakes by resource level (as previously described in Gunter et al. [28]), including the count of shelters in our sample that were included at each resource level.

Organizationally, most shelters were either private nonprofits (45%), private nonprofits with municipal contracts (31.7%), or public municipal agencies (23.3%). More often, shelters were open intake (46.7%), but managed-intake facilities were also common (36.7%). A smaller proportion of shelters in our study were limited admission (16.7%).

When describing the performances of shelters’ intervention programs as a summed ranking value of three performance metrics (i.e., number of intervention experiences, caregivers participating, and days of data collection), the average performance value for these programs was 62.2 (*Mdn* = 59.0, *SD* = 23.6, Range: 12–116).

Overall, 2327 dogs had a brief outing (1728) or temporary fostering stay (599) as part of this study. Because dogs could have more than one outing or stay, 3481 intervention experiences occurred: 2786 brief outings and 695 temporary fostering stays. Overall, 2367 caregivers participated, either as brief outing (1842) or temporary fostering (525) caregivers. These data represent all recorded experiences. A small portion of these experiences failed to meet study criteria (e.g., the outing duration was less than 1 hour or the fostering stay exceeded three days) and were removed from subsequent descriptive and statistical analyses.

#### 3.1.2. Dogs

The data of 27,901 dogs were used in our analyses: 1955 that participated in the brief outing and temporary fostering interventions, and 25,946 dogs that resided in the study shelters at the same time as the intervention dogs and served as controls. Over half of dogs in this dataset entered the shelter as strays (57.7%). Almost one-quarter of dogs were surrendered by their owner (16.9%) or were a failed adoption (6.4%), 14.1% were transferred from another facility, and 4.9% were brought to the shelter as part of a cruelty or neglect case. Males and females were relatively equally represented (males: 52.2%). Dogs were, on average, just over three years of age at the time of entering the study (*M* = 39.7 months, *SD* = 35.7, *Mdn* = 24, Range: 5.6–267.9) and weighed a mean of 18.4 kg (*SD* = 10.3, *Mdn* = 18.6, Range: 0.45–77.0).

A majority of dogs (87.8%) that received an outing or foster stay were available for adoption at the time of the study; however, 12.2% were not available, due to behavioral (4.2%), medical (4.0%), or other (4.0%) reasons (e.g., stray hold or awaiting transfer). The average length of stay for intervention dogs, excluding time out of the shelter during their brief outing or temporary fostering stay, was 35.1 days (*SD* = 42.3) with a median of 21.0 days. Dogs’ average length of stay pre-intervention was 32.7 days (*Mdn* = 14, *SD* = 52.4, Range: 0–623), and 9.9 days (*Mdn* = 5, *SD* = 14.6, Range: 0–157) after study participation. Dogs in our control condition had an average length of stay of 9.5 days (*Mdn* = 5, *SD* = 14.0, Range: 0–267).

At the end of the study, we found that outcomes for dogs in the interventions were mostly positive, although nearly a quarter (23.6%) remained in the care of their organization at the end of data collection (i.e., seven days after the final dog participated in the shelter’s intervention). Of those dogs still in the organization’s care, nearly all (98.7%) were residing in the shelter (as compared to a foster home). Almost two-thirds (65.2%) of dogs had been adopted into a home, 8.2% were transferred to another animal welfare organization, and less than one percent (0.9%) were returned to their owner. Less than two percent of dogs that participated in our interventions were euthanized for behavioral (1.1%), medical (0.3%), or capacity reasons (0.5%). Dogs that were returned to their owners were not included in the statistical analyses.

#### 3.1.3. Intervention

The average duration of an outing was 3.0 h (*SD* = 1.3, *Mdn* = 2.6, Range: 1–10) and 1.6 days (*SD* = 0.6, *Mdn* = 1.9, Range: 0.5–3 days) for a temporary fostering stay. Over three-quarters of dogs (77.1%) had only one outing or stay during the study, but 22.9% of dogs had two or more experiences. Overall, a total of 2437 brief outings and 496 temporary fostering stays were eligible for inclusion in these analyses.

During the 2934 intervention experiences that occurred as part of this study, a total of six bites were reported, representing <1% of all experiences. Most often, these bites were inflicted upon a person (five) while one incident was with another dog during a brief outing.

#### 3.1.4. Foster Caregivers

In total, 1842 brief outing and 408 temporary fostering caregivers were included in the statistical analyses. Caregivers were, on average, 39.0 years old (*SD* = 15.0) with a median age of 35.7 years. We found that members of the community, with no prior relationship to the shelter, were most often providing brief outings (47.5%) while shelter volunteers provided another 42.7% of outings. Conversely, volunteers more often temporarily fostered (45.4%) while community members provided 37.1% of temporary foster experiences. Additionally, shelter staff provided 7.7% of the study’s brief outings, and 11.3% of the temporary fostering stays. A small portion of outings (2.1%) and foster experiences (6.4%) were provided by caregivers who were not categorized by our study shelters.

Just over half (50.9%) of temporary foster caregivers had no resident dog in their home; with community members, this occurred much more often (70.1%; Table 2). When caregivers had a dog(s) living in their home, they most often introduced the dogs at the shelter prior to fostering (37.9%), followed closely by caregivers electing to keep the dogs separated during fostering (35.1%). Over a quarter of caregivers (27.0%) carried out the introduction between the dogs at their home.

For most intervention experiences analyzed in this study, caregivers did not adopt their dogs. Only 4.2% of outings and 12.0% of fostering stays resulted in an adoption by the caregiver providing the experience; however, this difference in caregiver adoptions between brief outings and temporary fostering stays was statistically significant: *χ*^2^ = 46.9, *p* < 0.001.

### 3.2. Intervention and Non-Intervention Dogs

#### 3.2.1. Intervention Impact on Shelter Outcomes

To better understand how brief outings and temporary fostering influenced dogs’ outcomes, we employed a series of multinomial logistic regression models. These models included the intervention impact (brief outing or temporary fostering versus controls) as well as the covariates of dog age, weight, sex, and intake type. To reduce outlier influence, weight was restricted from 1.36–45.36 kg, with values outside this range set to the described minimum or maximum value, and age capped at 150 months. These restrictions and cap for weight and age, respectively, were utilized in subsequent models in which these variables were included as covariates.

Our categorical dependent variable in these multinomial logistic regression models was adoption versus transfer to another agency, remaining at the shelter, euthanasia, or becoming lost or unexpectedly dying in the shelter. Because no dogs that received an intervention were lost or died in the shelter, this outcome was grouped with euthanasia.

**Figure 1:**
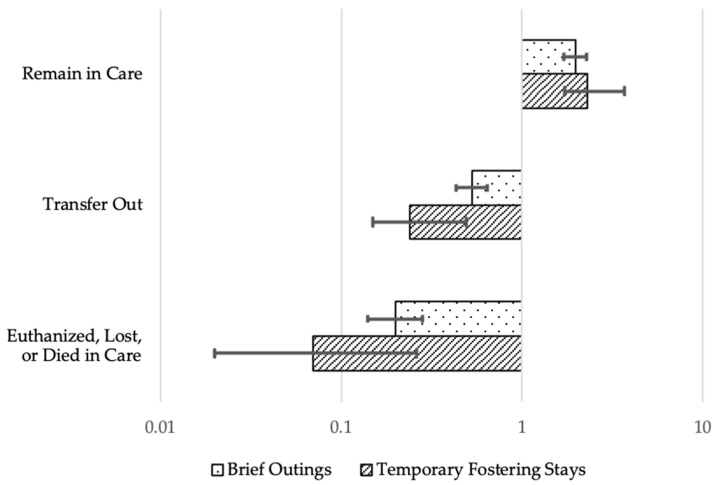
Relative risk (RR) ratios of adoption versus other outcomes for intervention dogs as compared to controls, adjusted for dog age, weight, sex, and intake type.

As represented in Figure 1, we found that dogs that experienced either intervention were less likely to be euthanized, become lost or die at the shelter (BO/RR = 0.20, 95% CI [0.14, 0.28]; TF/RR = 0.07, 95% CI [0.02, 0.21]), or be transferred to another animal welfare organization (BO/RR = 0.53, 95% CI [0.43, 0.64]; TF/RR = 0.24, 95% CI [0.15, 0.40]) than be adopted when compared to non-intervention dogs, controlling for other factors. As such, when dogs experienced a brief outing or a temporary fostering stay, they were 5.0 and 14.29 times more likely, respectively, to be adopted than euthanized as compared to dogs that did not receive these interventions. Intervention dogs were also more likely to remain in the care of their shelter at the end of the study than be adopted when compared to non-intervention dogs (BO/RR = 1.97, 95% CI [1.70, 2.26]; TF/RR = 2.30, 95% CI [1.72, 3.08]). Full model results are reported in Table S1 (Supplementary Materials).

#### 3.2.2. Intervention Impact on Length of Stay

To assess the impact of brief outings and temporary fostering on dogs’ time living in the shelter, we carried out two multilevel model analyses. These models included fixed effects for intervention and characteristics about the dog (i.e., sex, weight (restricted), age (capped), and intake type) as well as a random intercept for the shelter to estimate the intervention’s effect on length of stay (log-transformed) amongst intervention and non-intervention dogs that were adopted.

In the intervention models described in Table 3, we found that dogs that were heavier (*p* < 0.001) or older (*p* = 0.003) had longer lengths of stay. For dogs that experienced either intervention, they had, on average, longer lengths of stay than non-intervention dogs (*p* < 0.001). Furthermore, intervention dogs that arrived as a stray, part of a cruelty or neglect case, or were transferred from another organization had longer lengths of stay than non-intervention dogs that were owner-surrendered or returned after a failed adoption (*p* < 0.001). In our temporary fostering model, we found that female dogs had shorter lengths of stay than males, even when accounting for the other covariates (*p* = 0.002).

### 3.3. Intervention Dogs

#### 3.3.1. Duration of Brief Outings and Temporary Fostering Stays

To identify factors associated with outing duration, we utilized a multilevel model to examine the effects of dog characteristics (i.e., sex, weight (restricted), age (capped), intake type), caregiver characteristics (i.e., age, type), a dog bite to a human or other dog during the outing as well as a random intercept for shelter on the duration of outings. We found significant effects of dog weight and caregiver type. Dogs of greater weight had significantly shorter outings than dogs of lesser weight, *t*(2203) = −2.0, *p* = 0.042, while caregivers who were volunteers at the shelter were more likely to take dogs on longer outings than caregivers from the community, *t*(1625) = 2.7, *p* = 0.006, accounting for all other covariates.

To identify factors associated with the duration of temporary foster care, we utilized a multilevel model to examine the effects of dog characteristics (i.e., sex, weight (restricted), age (capped), intake type), caregiver characteristics (i.e., type, age, previous fostering experience), a bite to a person during the experience, and number of resident dogs in the caregiver’s home as well as a random intercept for shelter on fostering duration. Only one variable, the occurrence of a human bite, predicted significantly shorter temporary fostering stays, *t*(397) = −3.06, *p* = 0.002, accounting for all other covariates. Full model results are reported in Table S2 (Supplementary Materials).

#### 3.3.2. Shelter Outcomes by Intake Type

Using only the data from our intervention dogs, we conducted two multinomial logistic regression analyses with covariates to assess the effect of intake type on dog outcomes. Adoption was our reference category for the outcome variable, and dogs that were surrendered by their owners or returned by their adopters was our reference category for the intake type predictor variable. Other possible outcomes for intervention dogs include transfer to another organization, remaining in care (at the shelter or in foster care), euthanasia, or becoming lost or dying in the shelter. The covariates of dog age (capped), weight (restricted), and sex were used. In our temporary fostering analysis, the number of resident dogs in the caregiver’s home was included. Dog counts by intervention, intake type, and shelter outcome are provided in Table 4.

Among dogs that had a brief outing, we found that stray dogs were more likely to be transferred out to another agency (BO/RR = 2.44), or euthanized (BO/RR = 3.86), than be adopted as compared to owner-surrendered and returned dogs, controlling for other factors (Table 5). Dogs that were transferred into the shelter and had a brief outing were less likely to be transferred out again (BO/RR = 0.22) than be adopted as compared to dogs that were owner-surrendered or returned, controlling for other factors.

For cruelty and neglect dogs, those that experienced a brief outing during their shelter stay were more likely to be transferred to another facility (BO/RR = 4.49) and more likely to be euthanized (BO/RR = 11.98) than adopted as compared to dogs that were surrendered by their owner or returned, controlling for other factors.

The relative risk ratios and confidence intervals estimated with the multinomial logistic regression model for temporarily fostered dogs (as described in Table 6) should be interpreted with caution. As shown in Table 4, some predictor and dependent variable categories were rare. Although the model met convergence criteria, the presence of extremely small cell counts limits our abilities to properly estimate parameter variability. However, further grouping of shelter outcome categories to address low counts (e.g., grouping dogs that were transferred out with those that were lost, died in care, or euthanized) would have created unmeaningful groups. As such, these categories remain as described, despite their low prevalence. Nevertheless, these multinomial logistic regression results provide directional understanding of the relationships between intake type and outcome.

We found that stray dogs that were temporarily fostered were more likely to be transferred (TF/RR = 22.20) than adopted and less likely to be euthanized (TF/RR < 0.001) as compared to owner-surrendered and returned dogs, controlling for other factors. Dogs from cruelty and neglect cases that experienced a temporary fostering stay were less likely to be euthanized (TF/RR = 0.83) or transferred out (TF/RR < 0.001) than be adopted as compared to owner-surrendered/returned dogs, controlling for other factors.

For dogs that were temporarily fostered by a caregiver, we found that more resident dogs in their home corresponded to a higher likelihood of the fostered dog being transferred out of the shelter (TF/RR = 1.94) and a much lower likelihood of being euthanized (TF/RR < 0.001).

#### 3.3.3. Post-Intervention Length of Stay

To better appreciate how a brief outing or temporary fostering stay may have influenced dogs’ length of stay in the shelter after receiving the intervention, we employed multilevel modeling with fixed effects for dog sex, weight (restricted), age (capped), and intake type, and number of resident dogs in the caregiver’s home (for the temporary fostering model only) as well as a random intercept for the shelter to estimate the effect that these interventions had on post-intervention length of stay (log-transformed) among dogs that had a shelter outcome in our intervention groups. Log-transformed length of stay was our dependent variable.

For these dogs, we found that a dog’s weight was significantly related to longer lengths of stay post-intervention for dogs that experienced a brief outing, *t*(833) = 3.19, *p* = 0.001, or temporary fostering stay, *t*(220) = 3.60, *p* = 0.001. That is, as the weight of the dog increased, so did their time in the shelter post-intervention. For dogs that were temporarily fostered, their age also positively predicted longer lengths of stay after fostering, *t*(215) = 3.7, *p* = 0.0003. Full results of this model are reported in Table S3 (Supplementary Materials).

### 3.4. Overall Intervention Performance

We found that shelters with higher percentages of caregivers who were community members were more likely to have higher performing programs, *t*(45) = 4.27, *p* < 0.001, controlling for intervention type and other covariates. Additionally, public municipal agencies were more likely to have lower performing programs when compared to private, nonprofit organizations with municipal contracts, *t*(45) = −2.08, *p* = 0.044. Shelters with more resources were likely to have higher program performances, *t*(45) = 2.27, *p* = 0.028.

No other variables included in the model significantly predicted intervention performance. We also tested two interactions in our model, intervention type by proportion of volunteers who were foster caregivers as well as intervention type by proportion of community caregivers, but neither interaction was statistically significant, indicating that the effect of the proportion of volunteers or community members on program performance did not differ by intervention type. Table 7 describes the main effects and interactions that were tested in the ordinary least squares regression model of program performance.

## 4. Discussion

Our investigation found that interventions consisting of either a brief outing or temporary stay in a caregiver’s home resulted in shelter dogs being adopted more often as compared to dogs in shelters that did not receive these interventions. Dogs that participated in these interventions were also less likely to be transferred to another facility for placement. Adoptions by caregivers were infrequent but occurred more often after an overnight stay than an outing.

Our findings add to a growing body of fostering literature, including work by Ferhinger [15] and Gunter et al. [16,21], that has investigated the proximal effects of human interaction provided outside of the animal shelter on the welfare of shelter-living dogs. Previous studies found that overnight stays of any duration (one, two, or three nights) reduced dogs’ cortisol levels and increased rest whereas brief outings did not, and as such, these interventions do not have the same effects on dogs’ immediate welfare and recommendations for their usage have differed [20].

The current study provides evidence about the distal benefits of both brief outings and temporary fostering stays, most importantly their influence on shelter dog adoptions. Simply put, dogs leave animal shelters alive more often when they have an outing of just a few hours or stay in a home with a person, five or over 14 times so, respectively. Moreover, these dogs typically had longer shelter stays prior to experiencing these interventions. Previously, Patronek and Crowe [27] found a positive effect of canine foster caregiving, increasing the likelihood of live outcomes by five to over 20 times, depending on a dog’s intake type into the shelter, as compared to dogs that did not enter foster care.

Nevertheless, dogs in our study that were surrendered by their owners or returned by adopters, and then temporarily fostered, were more likely to be euthanized than temporarily fostered dogs that arrived as strays or were part of cruelty or neglect investigations. During temporary fostering stays, it is possible that caregivers’ observations coincided with behavioral concerns about these dogs that were expressed by their previous owners and played a role in the dogs’ negative outcomes. Prior work by Duffy et al. [31] and Stephen and Ledger [32] found that relinquishing owners’ reports about their dogs’ aggression toward strangers were significantly correlated to reports by the dogs’ new adopters about the same behavior. Nevertheless, future research with a larger sample size of temporarily fostered dogs would allow for more accurate parameter estimation concerning these intake types and outcomes.

Conversely, owner-surrendered and returned dogs that left the shelter on brief outings were more likely to be adopted as compared to their stray and cruelty/neglect counterparts. Additionally, we found that when dogs were temporarily fostered in homes with multiple resident dogs, they were more likely to be adopted and twice as likely to be transferred out of the shelter for placement. It is possible that a dog’s friendliness with other dogs may be related to these outcomes, a factor that has been previously reported as influential in dog adoptions and relinquishments to the shelter [33,34,35,36].

With regards to caregivers adopting their fostered dogs, it is possible that such decisions could be influenced by the duration of the caregiving experience. Here, we found that brief outings had the lowest percentage of adoptions by a caregiver, 4%, while 12% of temporary fostering stays resulted in adoption. During the pandemic, Gunter et al. [28] found that adult dogs were adopted by their caregivers in 18% of foster experiences, which typically involved much more time in the caregiver’s home. However, Gunter et al. [28] also found that intention to adopt (i.e., trial adoption programs), the type of foster caregiver, and number of dogs in a caregiver’s home influenced adoption likelihood as well. Thus, it seems that while shelters will achieve better distal welfare for dogs by utilizing brief outing and temporary fostering interventions, the adoption of these dogs will likely not be by the caregivers themselves.

Another aspect of dogs’ welfare in the shelter is the time spent in the organization’s care awaiting an outcome. Few experimental interventions in the shelter have been shown to reduce dogs’ time living in the shelter, while a greater number of interventions have been identified that increase adoption likelihood [23,24,25,26,27]. In the present study, we found that dogs that participated in either intervention had longer lengths of stay and more often remained in the care of the shelter at the end of study compared to non-intervention dogs. More specifically, we found that dogs that received a brief outing or fostering stay had lengths of stay between 32 and 34 days, respectively, prior to the intervention, while dogs that did not experience either intervention resided in the shelter for just 10 days.

It is conceivable that intervention dogs’ longer lengths of stay before their brief outings or fostering stays may have been related to qualities about the dogs themselves; specifically, characteristics that negatively impact adoption likelihood, such as a dog’s morphology [37] or social behavior during meet-and-greets [38]. Animal shelters are often encouraged to use brief outing or temporary fostering programs for adoption promotion, particularly those dogs that have resided in the shelter for extended periods of time [39]. As such, intervention dogs’ longer lengths of stay may not be related to the intervention’s effect, but, instead, were a consideration when shelter staff selected dogs for outings and fostering stays.

After the intervention, shelters would have had more information about these dogs and might have felt better informed about their viability as adoption candidates. Supportively, we found that placement of intervention dogs was more likely to occur through adoption, rather than transferring them to another organization for placement, which may be indicative of the shelter’s continued investment or a lack of perceived attractiveness by other organizations. Additionally, no intervention dogs were lost or died unexpectedly in the shelter during the study. After their brief outing or temporary fostering stay, dogs waited an average of just 10 days to be adopted, which is considerably shorter than their lengths of stay beforehand. Such a finding suggests that ultimately, these dogs’ distal welfare was positively impacted by the interventions.

Furthermore, highly desirable dogs may have not resided long enough in the shelter to participate in our interventions, which could account for the difference in length of stay observed between the two groups. As such, future studies may consider matching dogs on multiple morphological and behavioral variables to further understand the effects of these interventions.

Age has also been shown to influence time to adoption from foster care during the pandemic [28] as well as likelihood of return after adoption [35]. Across our dataset, we found that heavier and older dogs stayed longer in the shelter awaiting an outcome, and the effect of weight on length of stay persisted post-intervention. The effects of age and weight on shelter dog outcomes have been previously reported, most recently by Cain et al. [7,40]. In our study, we found that a dog’s weight also influenced the duration of their brief outing, such that larger dogs received shorter outings than smaller dogs.

One possible explanation for this effect of weight on outing duration may be related to the force exerted by larger dogs while on-leash. Shih et al. [41] found that dogs of greater size and weight exhibit more tension on-leash, and increased tension on the leash has been shown to negatively impact volunteers’ satisfaction walking shelter dogs [42]. Thus, it is possible that caregivers on outings with larger dogs may have been less satisfied due in part to an inability to handle their dogs, resulting in earlier returns to the shelter. In future studies, examining the effects of dog walking equipment on outing duration and caregiver satisfaction may elucidate ways that these experiences can be improved, which could increase the distal benefits of this intervention, particularly for larger dogs that often reside in shelters longer.

Caregivers in the present study were slightly older than those that fostered dogs during the pandemic [28], but both studies found that caregivers are usually early middle-aged adults. Previously, foster caregivers have been reported to more often be pet owners [43]. However, we found a greater proportion of temporary foster caregivers in this study and those that were pandemic caregivers did not own a dog [28]. While caregiving opportunities in these studies may have been more appealing to non-dog-owners, it is also possible that these studies’ larger datasets, which captured all caregivers participating in the interventions versus only those caregivers willing to complete a survey, may have led to this difference in findings. As we did not collect the pet-owning status of brief outing caregivers (because the shelter dogs were not residing in their homes), we are unable to describe this attribute further. Worth noting, however, is that Ackermann et al. [43] did identify that a key difference in the motivation of early middle-aged foster caregivers, as opposed to younger and older caregivers, was not wanting the responsibility of pet ownership, which may explain the lack of dog ownership by caregivers that we observed in this study.

In their exploration of fostering during the pandemic, Gunter et al. [28] found that members of the community with no prior relationship to the shelter played the largest role in foster caregiving. In this study, we also found that community involvement was influential. Specifically, brief outing and temporary fostering programs that had greater proportions of community caregivers providing experiences were higher performing as defined here by the number of intervention experiences the shelter provided, foster caregivers they engaged, and days needed to carry out the study. Across our shelters, members of the community were more often engaged in brief outing programs whereas volunteers had a greater presence in shelters’ temporary fostering efforts.

The importance and impact of community engagement in animal sheltering, such as strategies used by animal control and field service officers, have been previously described by Moss et al. [44]. Our findings suggest that not only do interventions that engage individuals beyond the shelter’s volunteers and staff lead to more successful programs, but these shorter-duration fostering interventions can significantly impact outcomes for dogs. As such, we believe that removing barriers to community participation in these programs can save the lives of more dogs awaiting adoption in United States animal shelters.

Prior work has identified that individuals are hesitant to foster shelter animals because of the emotional attachment and time involved in caregiving as well as limitations caused by their own pets and housing status [45]. Brief outings address these concerns as they are of a minimal duration and do not require housing the dog. As evidenced with our data, this particular intervention may be a powerful engagement tool, particularly as animal shelters struggle to recruit and retain foster caregivers [46].

Social exchange theory, as described by Schafer [47] in relation to volunteers, may be a better way to understand the motivations of foster caregivers, and provide us insights into how brief outings and temporary foster care could shape greater community involvement. Foster caregiving is high-stakes volunteerism [28,48], and it is likely that as the duration of caregiving increases, so do the costs to the caregiver and risk (and reward) of emotional attachment. In order to address these concerns, we posit that shorter-duration foster care as studied here should be commonly practiced so that the rewards of caregiving easily exceed the costs, especially for first-time caregivers. As rewards are repeatedly experienced by caregivers through brief outings and temporary fostering stays, riskier fostering opportunities of longer durations could be embarked upon. Such an approach may address the emotional stress of this type of volunteerism, and retention issues that are often experienced by animal welfare organizations [43,46,49,50].

During this study’s nearly three thousand intervention experiences, dog bites were exceedingly rare, but when they did occur, they more often involved the dog biting a person versus another dog. Not surprisingly, we found that such events were related to shorter fostering stays in homes, likely indicative of their negative effect on the caregiver’s experience. Bites to humans and dogs were also rarely reported by Gunter et al. [28] in over 2500 fostering experiences of longer durations. With the relatively low risk to human safety associated with foster caregiving of varying durations and the benefits of these programs on shelter dog outcomes, it is not surprising that organizations with foster care programs have higher rates of live release and lower returns of adopted dogs [51].

With regards to the evaluation of the shelters’ brief outing and temporary fostering programs, we did find that municipal shelters typically had lower performing programs. During the pandemic, Gunter et al. [28] also observed that municipal shelters’ utilization of foster care was lower and more quickly returned to pre-pandemic levels compared to shelters that were either private nonprofits or private nonprofit organizations with municipal contracts. Furthermore, in this study, we found that shelters with greater resources had higher performing programs, highlighting the importance of human and financial resources in animal welfare. Thus, while brief outings and temporary fostering programs should be explored by a variety of organizations given their potential impact on dog welfare, it is likely that municipal agencies will need to provide additional support to staff while better-resourced shelters may be able to implement these programs more easily with greater effect.

When considering the limitations of our study, it is likely that the requirements of programmatic training, implementation, and data collection were barriers to participation for lower-resourced shelters, undermining our efforts to enroll a diverse sample of animal shelters operating in the United States. The average live release rate for shelters in the current study was above 90%, which is high, but comparable with data reported by industry organizations [52]. Additionally, it is unknown how our findings may generalize to animal shelters in other countries, particularly localities where foster caregiving is not as commonplace.

In their study, Gunter et al. [28] reported similar resource levels of participating shelters, and in that investigation, shelters were not required to attend training or implement a specific intervention beyond placing dogs in foster care. Nevertheless, the obligation of placing dogs in foster care in and of itself could have been an impediment to participation across both studies as nearly all participating shelters had existing canine foster care programs at the time of study enrollment.

Data collection about dogs receiving these interventions was overseen by our research team; however, we relied upon shelter management systems for data about non-participating dogs. While such systems are routinely utilized in research about dogs in animal shelters, the availability of data and completeness of dogs’ records likely differed between our control and intervention conditions. Moreover, longer data collection periods following the interventions would have likely resulted in fewer intervention dogs remaining in care at the end of the study, improving our outcome predictions.

It is possible that not all dogs at these shelters were eligible to participate in the interventions that were tested. The majority of dogs that experienced a brief outing or temporary fostering stay were available for adoption with a small proportion that were unavailable due to behavioral or medical concerns. Nevertheless, dogs with greater safety concerns related to their behavior were likely not selected for participation in our interventions but remained available for adoption or were euthanized soon after intake, which may have contributed to the higher likelihood of adoption in our intervention groups and shorter lengths of stay for control dogs. As described in previous studies about brief outings and temporary fostering interventions [16,21], shelter staff often do not enroll dogs with histories of aggression in these types of programs, which may explain the low incidents of human and dog bites that were reported.

## 5. Conclusions

This study demonstrates that brief outings and temporary fostering stays result in a greater likelihood of adoption for dogs in animal shelters when compared to dogs that do not experience these interventions. Adoptions were seldom by the caregivers themselves, although when this did occur, it was more often after a temporary fostering stay. Nevertheless, dogs that participated in either intervention had longer lengths of stay and were more often awaiting adoption at the end of the study as compared to non-intervention dogs, although this difference in length of stay was present prior to study enrollment and may be related to morphological and behavioral qualities of the intervention dogs.

When intervention programs of either type had greater percentages of community members participating, these programs were higher performing. Brief outing caregivers were more often individuals from the community whereas shelter volunteers were more involved in temporary foster care. As such, brief outings may be a means to address the caregiver recruitment issues faced by animal welfare organizations.

In all, shorter-duration fostering interventions as studied here may better balance the costs and rewards involved with this type of high-stakes volunteerism and assist in the retention of foster caregivers. However, shelter resources play a role in the programmatic success of these interventions. Organizations need to provide the human and financial means necessary to operate these programs in order to positively impact the dogs in their care.

## Figures and Tables

**Table 1:** Shelter resource levels and associated annual budgets, animal intake numbers, and resources per animal.

Resource Level	*M, Mdn* Annual Budget	Annual Budget Range (Min–Max)	*M, Mdn* Animal Intake	Animal Intake Range (Min–Max)	*M, Mdn* Resources per Animal	Resources per Animal Range (Min–Max)	Shelters in This Dataset
Very Low	1.9 M, 965 K	200 K–4.3 M	10,867, 7215	826–29,595	209, 242	118–276	11
Low	4.2 M, 3 M	1.7 M–13 M	10,016, 8118	3873–32,788	406, 403	370–439	9
Moderately Low	2.2 M, 1.8 M	730 K–4.3 M	4077, 3241	1256–8916	562, 572	473–648	10
Moderate	2.3 M, 1.2 M	300 K–6.9 M	3021, 1494	408–8834	759, 749	699–845	11
High	8.4 M, 6.6 M	1.5 M–23 M	8244, 6377	1498–23,093	1028, 1022	918–1105	6
Very High	7.4 M, 9.9 M	386 K–14 M	4299, 3478	241–8117	1849, 1647	1480–2852	11

Note. Abbreviations: Millions (M), Thousands (K). Average Annual Budget and Average Resources Per Animal and their associated ranges are in USD. Resources Per Animal is an estimated value calculated by dividing a shelter’s annual budget by the previous year’s animal intake. Resource Level calculations are not included for two organizations that were unable to provide yearly animal intake numbers.

**Table 2:** Temporary fostering caregivers and number of resident dogs living in their homes.

		Percent of Caregivers (%)				
		Number of Resident Dogs in Home				
Type of Caregiver	*n*	0	1	2	3	Not Reported
Community Member	174	70.1	21.3	5.8	1.7	0.6
Volunteer	173	53.8	15.6	11.0	2.9	19.7
Staff	42	28.6	26.2	23.8	19.1	2.4
Not Reported	19	0	0	0	0	100
Total	408	50.9	19.1	12.5	3.8	13.7

**Table 3:** Fixed effects of brief outing and temporary fostering interventions and model covariates on dogs’ length of stay.

	Brief Outing					Temporary Fostering				
Fixed Effect	Est	*SE*	*df*	*t*	*p*	Est	*SE*	*df*	*t*	*p*
(Intercept)	1.46	0.08	53	18.07	<0.001	1.60	0.05	139	29.72	<0.001
Intervention vs. Population	0.88	0.03	6235	26.81	<0.001	0.89	0.05	4123	16.30	<0.001
Female vs. Male	−0.01	0.02	6291	−0.28	0.781	−0.11	0.03	4154	−3.71	0.002
Dog Weight	0.01	0.00	6301	17.34	<0.001	0.01	0.00	4156	9.83	<0.001
Dog Age	0.00	0.00	6298	6.87	<0.001	0.00	0.00	4154	2.98	0.003
Stray vs. Owner Surrender	0.48	0.03	6316	16.72	<0.001	0.53	0.04	4098	14.99	<0.001
Cruelty/Neglect vs. Owner Surrender	0.56	0.06	6308	9.72	<0.001	0.94	0.11	4128	8.38	<0.001
Transfer In vs. Owner Surrender	0.13	0.03	6309	4.18	<0.001	0.18	0.04	4118	4.57	<0.001

**Table 4:** Counts of dogs by intervention, intake type, and outcome.

	Outcome Counts of Brief Outing Dogs				Outcome Counts of Temporary Fostering Dogs			
Intake Type	Adoption	Remain in Care	Transfer Out	Euthanized, Lost, or Died in Care	Adoption	Remain in Care	Transfer Out	Euthanized, Lost, or Died in Care
Owner Surrender	341	147	40	6	122	27	1	2
Stray	319	143	93	25	83	21	13	0
Transfer In	275	97	7	3	134	19	3	1
Cruelty/Neglect	11	7	5	2	1	3	0	0

**Table 5:** Dog outcome relative risk (RR) ratios with 95% lower and upper confident limits (LCLs and UCLs) for dogs that experienced a brief outing.

	Shelter Outcomes for Brief Outing Dogs								
	Remain in Care vs. Adopted			Transfer Out vs. Adopted			Euthanized, Lost, or Died in Care vs. Adopted		
Covariates	RR	LCL	UCL	RR	LCL	UCL	RR	LCL	UCL
(Intercept)	0.19	0.12	0.29	0.18	0.09	0.35	0.01	0.001	0.03
Female vs. Male Dogs	1.06	0.83	1.34	0.86	0.59	1.26	0.56	0.26	1.22
Dog Weight (kg)	1.02	1.01	1.04	0.97	0.95	0.99	1.07	1.02	1.12
Dog Age (months)	1.01	1.00	1.01	1.01	1.00	1.01	0.99	0.97	1.01
Stray vs. Owner Surrender	1.16	0.87	1.54	2.44	1.59	3.74	3.86	1.52	9.84
Cruelty/Neglect vs. Owner Surrender	1.60	0.60	4.23	4.49	1.46	13.81	11.98	2.12	67.78
Transfer In vs. Owner Surrender	0.98	0.71	1.34	0.22	0.09	0.50	0.77	0.19	3.16

Note. Adoption is the outcome reference category in this analysis, and dogs that were surrendered by their owners or returned by their adopter is the comparison group for intake type. An RR value > 1 indicates that the comparison outcome is that many times more likely to occur instead of adoption as the predictor value increases (or for the comparison intake type than for owner-surrendered or returned dogs). With an RR value < 1, divide 1 by the RR value to calculate how many times more likely adoption is to occur as the predictor value increases (or for the comparison intake type than for owner-surrendered or returned dogs).

**Table 6:** Dog outcome relative risk (RR) ratios with 95% lower and upper confident limits (LCLs and UCLs) for dogs who experienced a temporary fostering stay.

	Shelter Outcomes for Temporarily Fostered Dogs								
	Remain in Care vs. Adopted			Transfer Out vs. Adopted			Euthanized, Lost, or Died in Care vs. Adopted		
Covariates	RR	LCL	UCL	RR	LCL	UCL	RR	LCL	UCL
(Intercept)	0.17	0.06	0.51	0.002	0	0.03	0.02	0.001	0.99
Number of Resident Dogs	0.82	0.56	1.21	1.94	1.18	3.19	<0.001	<0.001	<0.001
Female vs. Male Dogs	0.92	0.50	1.68	1.31	0.44	3.94	<0.001	<0.001	<0.001
Dog Weight (kg)	1.01	0.98	1.04	1.06	0.99	1.13	1.04	0.92	1.18
Dog Age (months)	1.00	0.10	1.01	0.99	0.97	1.01	0.10	0.97	1.03
Stray vs. Owner Surrender	1.10	0.51	2.34	22.20	2.72	180.90	<0.001	<0.001	<0.001
Cruelty/Neglect vs. Owner Surrender	6.35	0.34	117.10	<0.001	<0.001	<0.001	0.83	0.83	0.83
Transfer In vs. Owner Surrender	0.50	0.23	1.10	2.41	0.24	24.58	0.52	0.04	6.42

Note. Adoption is the outcome reference category in this analysis, and dogs that were surrendered by their owners or returned by their adopter is the comparison group for intake type. An RR value > 1 indicates that the comparison outcome is that many times more likely to occur instead of adoption as the predictor value increases (or for the comparison intake type than for owner-surrendered or returned dogs). With an RR value < 1, divide 1 by the RR value to calculate how many times more likely adoption is to occur as the predictor value increases (or for the comparison intake type than for owner-surrendered or returned dogs).

**Table 7:** Effects of shelter characteristics on the performance of intervention programs.

Effect	Est	*SE*	*t*	*p*
(Intercept)	37.04	13.68	2.71	0.010
Intervention (Temporary Fostering vs. Brief Outing)	−50.05	34.55	−1.45	0.154
Percent Volunteers	22.44	15.85	1.42	0.164
Percent Community Members	62.70	14.68	4.27	<0.001
Shelter Resources	0.01	0.00	2.27	0.028
Municipal vs. Nonprofit w/Municipal Contracts	−12.85	6.20	−2.08	0.044
Nonprofit vs. Nonprofit w/Municipal Contracts	−8.38	6.51	−1.29	0.205
Managed vs. Open Admission	−1.17	6.19	−0.19	0.851
Limited vs. Open Admission	−10.73	8.85	−1.21	0.232
Intervention Type by Percent Volunteers	29.77	40.99	0.73	0.471
Intervention Type by Percent Community Members	5.30	36.58	0.15	0.885

## Data Availability

The data presented in this study are openly available in the Virginia Tech Data Repository.

## Supplementary Materials

The following supporting information can be downloaded at: https://www.mdpi.com/article/10.3390/ani13223528/s1.
