# Supplementary material for: The Influence of Brief Outing and Temporary Fostering Programs on Shelter Dog Welfare
Source: Animals (Basel). 2023 Nov 15;13(22):3528. doi: 10.3390/ani13223528 (PMC10668817; doi:10.3390/ani13223528)
Supplement: Supplementary file 1 [file animals-13-03528-s001.zip › animals-2671651-supplementary.pdf]

Table S1. Relative risk (RR) ratios and 95% lower and upper confidence limits (LCL & UCL) for dogs that experienced a brief outing or temporary fostering stay on shelter outcomes

	**Shelter Outcomes**								
	Remain in Care vs. Adopted			Transfer Out vs. Adopted			Euthanized, Lost, or Died in Care vs. Adopted		
**Covariates**	RR	LCL	UCL	RR	LCL	UCL	RR	LCL	UCL
(Intercept)	0.05	0.03	0.06	0.23	0.18	0.28	0.06	0.05	0.08
Temporary Fostering Stay vs. Control	2.30	1.72	3.08	0.24	0.15	0.40	0.07	0.02	0.21
Female vs Male Dogs	0.89	0.73	1.09	0.94	0.81	1.09	0.70	0.58	0.83
Dog Weight (kg)	1.04	1.03	1.05	0.98	0.97	0.99	1.04	1.03	1.05
Dog Age (months)	1.00	1.00	1.01	1.01	1.01	1.01	1.01	1.01	1.01
Stray vs. Owner Surrender	1.16	0.92	1.46	1.55	1.32	1.82	1.52	1.25	1.85
Cruelty/Neglect vs. Owner Surrender	5.88	3.87	8.94	2.42	1.62	3.59	4.20	2.77	6.36
Transfer In vs. Owner Surrender	0.49	0.36	0.67	0.11	0.07	0.17	0.20	0.13	0.30
(Intercept)	0.07	0.05	0.08	0.23	0.19	0.28	0.03	0.02	0.04
Brief Outing vs Control	1.97	1.70	2.26	0.53	0.44	0.65	0.20	0.14	0.28
Female vs Male Dogs	0.91	0.81	1.03	1.01	0.91	1.13	0.79	0.68	0.92
Dog Weight (kg)	1.05	1.04	1.06	1.00	0.99	1.00	1.06	1.06	1.07
Dog Age (months)	1.01	1.00	1.01	1.01	1.01	1.01	1.01	1.01	1.01
Stray vs. Owner Surrender	1.49	1.30	1.71	1.58	1.40	1.79	2.29	1.92	2.72
Cruelty/Neglect vs. Owner Surrender	1.28	0.94	1.72	1.45	1.13	1.87	2.41	1.77	3.28
Transfer In vs. Owner Surrender	0.44	0.36	0.53	0.10	0.07	0.13	0.23	0.16	0.32

Table S2. Duration of brief outings and temporary fostering stays

**Fixed Effect** (Brief Outing Model)	Est	*SE*	*df*	*t*	*p*
(Intercept)	2.98	0.15	199.26	19.38	< .001
Female vs. Male	0.03	0.05	2203.48	0.66	0.508
Dog Weight (kg)	-0.01	0.003	2202.88	-2.04	0.042
Dog Age (months)	0.001	0.001	2209.02	1.02	0.309
Stray vs. Owner Surrender	0.03	0.06	2221.65	0.52	0.602
Cruelty/Neglect vs. Owner Surrender	-0.13	0.24	2220.04	-0.55	0.586
Return vs. Owner Surrender	0.01	0.10	2210.68	0.13	0.900
Transfer In vs. Owner Surrender	0.07	0.08	2141.07	0.89	0.391
Staff vs. Community Member	-0.09	0.12	1866.21	-0.72	0.471
Volunteer vs. Community Member	0.22	0.08	1625.53	2.73	0.006
Dog Bite to a Person	-1.09	0.78	2193.22	-1.39	0.165
Dog Bite to Another Dog	-0.04	1.10	2189.84	-0.03	0.974
Caregiver Age (years)	-0.001	0.002	2219.29	-0.52	0.601

**Random Effect**			
Groups	Name	Variance	*SD*
Shelter ID	(Intercept)	0.32	0.57
Residual		1.19	1.09

**Fixed Effect** (Temporary Fostering Model)	Est	*SE*	*df*	*t*	*p*
(Intercept)	40.70	3.03	49.25	13.44	< .001
Female vs. Male	-1.07	0.79	399.49	-1.36	0.176
Dog Weight (kg)	-0.01	0.05	401.16	-0.30	0.767
Dog Age (months)	-0.01	0.01	400.09	-0.46	0.648
Stray vs. Owner Surrender	0.69	1.24	402.52	0.56	0.578
Cruelty/Neglect vs. Owner Surrender	5.20	5.61	396.91	0.93	0.355
Return vs. Owner Surrender	1.40	1.32	399.89	1.07	0.860
Transfer In vs. Owner Surrender	0.37	1.17	403.07	0.31	0.755
Number of Resident Dogs	-0.30	0.51	401.25	-0.58	0.560
Caregiver previously provided outing	0.70	1.04	400.78	0.67	0.504
Caregiver previously provided fostering stay	0.10	1.001	401.20	0.10	0.923
Staff vs. Community Member	-2.52	1.55	406.73	-1.62	0.105
Volunteer vs. Community Member	0.62	1.22	412.59	0.51	0.611
Dog Bite to a Person	-13.96	4.56	397.38	-3.06	0.002
Caregiver Age (years)	0.03	0.03	398.64	1.15	0.249

**Random Effect**			
Groups	Name	Variance	*SD*
Shelter ID	(Intercept)	99.52	9.98
Residual		56.54	7.52

Table S3. Post-intervention length of stay for dogs that experienced a brief outing or temporary fostering stay

**Fixed Effect** (Brief Outing Model)	Est	*SE*	*df*	*t*	*p*
(Intercept)	1.37	0.14	285.11	9.52	< .001
Female vs. Male Dogs	0.06	0.07	829.12	0.82	0.412
Dog Weight (kg)	0.01	0.004	834.23	3.43	0.001
Dog Age (months)	0.001	0.001	831.97	1.33	0.183
Stray vs. Owner Surrender	0.05	0.09	837.41	0.61	0.544
Cruelty/Neglect vs. Owner Surrender	0.15	0.32	832.62	0.46	0.643
Transfer In vs. Owner Surrender	0.03	0.10	819.11	0.35	0.730

**Random Effect**			
Groups	Name	Variance	*SD*
Shelter ID	(Intercept)	0.21	0.46
Residual		0.90	0.95

**Fixed Effect** (Temporary Fostering Model)	Est	*SE*	*df*	*t*	*p*
(Intercept)	0.88	0.27	143.92	3.21	0.002
Female vs. Male Dogs	0.001	0.13	216.91	0.01	0.996
Dog Weight (kg)	0.03	0.01	220.79	3.62	< .001
Dog Age (months)	0.01	0.002	215.64	3.82	< .001
Stray vs. Owner Surrender	-0.02	0.21	221.89	-0.08	0.934
Transfer In vs. Owner Surrender	-0.14	0.17	221.89	-0.85	0.397
Number of Resident Dogs	0.08	0.08	221.34	0.92	0.358

**Random Effect**			
Groups	Name	Variance	*SD*
Shelter ID	(Intercept)	0.22	0.47
Residual		0.92	0.96
